# Patient perspectives on considerations, tradeoffs, and experiences with multiple myeloma treatment selection: a qualitative descriptive study

**DOI:** 10.1186/s12885-022-10458-x

**Published:** 2023-01-19

**Authors:** Carrie Dombeck, Teresa Swezey, Juan Marcos Gonzalez Sepulveda, Bryce B. Reeve, Thomas W. LeBlanc, David Chandler, Amy Corneli

**Affiliations:** 1 Department of Population Health Sciences, Duke University School of Medicine, 215 Morris Street, Durham, NC 27701 USA; 2 Department of Medicine, Duke University School of Medicine, Durham, NC USA; 3 Duke Clinical Research Institute, Duke University School of Medicine, NC Durham, USA; 4 Duke Cancer Institute, Duke University School of Medicine, Durham, NC USA; 5 Amgen, Inc, Thousand Oaks, CA USA

**Keywords:** Multiple myeloma, Treatment selection, Tradeoffs, HRQoL, Qualitative research, Patients

## Abstract

**Background:**

Advances in multiple myeloma treatment and a proliferation of treatment options have resulted in improved survival rates and periods of symptom-free remission for many multiple myeloma patients. As a result, health-related quality of life (HRQoL) concerns related to myeloma treatments have become increasingly salient for this patient population and represent an important consideration guiding patients’ treatment choices. To gain an understanding of patients’ experiences with choosing myeloma therapies and explore the HRQoL concerns that are most important to them, we interviewed a diverse sample of US-based multiple myeloma patients about their treatment considerations.

**Methods:**

We conducted a qualitative descriptive study using in-depth interviews. Participants reflected on (1) the factors that were most important to them when thinking about multiple myeloma treatment and how these have changed over time, (2) how they might weigh the importance of treatment efficacy vs. side effects, (3) trade-offs they would be willing to make regarding efficacy vs. HRQoL, and (4) treatment changes they had experienced. Interviews were audio-recorded and transcribed, and narratives were analyzed using applied thematic analysis.

**Results:**

We interviewed 21 patients, heterogeneous in their disease trajectory and treatment experience. Participants were 36 to 78 years, 52% female, and 38% Black. Efficacy was named as the most important treatment consideration by almost two-thirds of participants, and over half also valued HRQoL aspects such as the ability to maintain daily functioning and enjoyment of life. Participants expressed concern about potential treatment side effects and preferred more convenient treatment options. Although participants stated largely trusting their clinicians’ treatment recommendations, many said they would stop a clinician-recommended treatment if it negatively impacted their HRQoL. Participants also said that while they prioritized treatment efficacy, they would be willing to change to a less efficacious treatment if side effects became intolerable.

**Conclusions:**

Our findings link to other reports reflecting considerations that are important to multiple myeloma patients, including the importance placed on increasing life expectancy and progression-free survival, but also the tension between treatment efficacy and quality of life. Our results extend these findings to a racially diverse US-based patient population at different stages in the disease trajectory.

## Background

Multiple myeloma is the second most common hematologic malignancy [1] and accounts for about 10% of all blood cancers [2]. In 2021, it is estimated that 34,920 new cases will be diagnosed in the United States, and about 12,410 deaths from myeloma will occur [3]. At a population level, multiple myeloma is slightly more prevalent in males [4] and has been found to occur twice as often in Black Americans than White Americans [5].

Recent years have seen rapid evolution in myeloma treatments [6], such that patients now have access to a wealth of treatment options [7] and have realized corresponding improvement in survival rates [8]. It is now common for myeloma patients to experience periods of symptom-free remission during which they continue to undergo maintenance therapy, although most patients eventually relapse, and the periods of remission shorten between each treatment cycle [9]. While multiple myeloma remains incurable, these advances have meant that many patients now experience it as a chronic disease, rather than as one that is rapidly terminal [10].

Improved survivorship has also given more salience to quality of life concerns [11, 12], as living longer has necessarily shifted patients’ focus away from worry about immediate mortality and towards the need to deal with disease-related symptoms and treatment side effects on an ongoing basis [13]. Cumulative toxicity and adverse effects related to treatment, as well as considerations related to treatment administration, thus increasingly serve to guide patients’ treatment choices [14, 15].

To gain an understanding of patients’ experiences with multiple myeloma and explore the values and issues that are most important to them, many researchers have turned to qualitative methods to document myeloma patients’ perspectives and experiences [16]. Qualitative studies have also been used to examine patient preferences for myeloma treatment options [17, 18]. However, the bulk of qualitative research into myeloma patients’ treatment preferences has been conducted with European patient samples; studies of US-based patient preferences are lacking. Further, none of these studies has purposefully sought to include non-White participants, aiming to obtain a sample more representative of myeloma distribution in the general population.

In this manuscript, we describe findings from qualitative interviews with diverse multiple myeloma patients in the US on considerations, including health-related quality of life (HRQoL) concerns, that may affect their decisions to change or continue taking multiple myeloma therapies. The findings were subsequently used to develop a discrete-choice experiment (DCE) survey to estimate patient tradeoff preferences for features of multiple myeloma therapies and evaluate patient tolerance for issues associated with treatment continuation or change; the DCE findings will be reported elsewhere.

## Methods

### Study design and participants

We conducted a qualitative descriptive study [19, 20] using in-depth interviews (IDIs). Study participants were English-speaking US adults aged 18 years or older with a self-reported or physician-confirmed diagnosis of multiple myeloma. Participants were recruited via email invitations sent to members of the multiple myeloma registry within the Cancer Support Community (CSC), an online panel of patients (https://www.cancersupportcommunity.org/registry) who have agreed to participate in research, and via clinician referral from a single center Adult Blood and Marrow Transplant Clinic in North Carolina. Among eligible participants, we purposefully selected [21] and invited individuals for interviews based on desired background and demographic characteristics (i.e., relapse status, race, and gender), with a specific goal of recruiting Black patients to approximate the proportions of Black multiple myeloma patients in the US general population. The interview sample size was informed by the concept of informational power [22], which outlines five factors to consider when determining sample sizes for qualitative research, such as the scope of the study aim and specificity of participant experiences.

### Data collection

Two trained qualitative interviewers conducted one-on-one interviews by telephone from July 9, 2019 to June 18, 2020. Questions explored participants’ perspectives on and experiences with multiple myeloma and its treatments, with a focus on decisional considerations and quality of life concerns (see Appendix for selected questions from the interview guide that informed this analysis). We asked participants to describe the course of their multiple myeloma treatment, the factors that were most important to them when thinking about multiple myeloma treatment, and how these have changed over time. Participants who had experienced a relapse since diagnosis and treatment initiation were asked to focus only on the factors that were most important to them since relapsing. When discussing the factors that were of greatest importance to them, participants were also asked to consider whether they would want a cancer treatment with fewer side effects even if less effective than other treatments, versus the most effective treatment even with many side effects. Further, we asked participants about trade-offs they might choose to make regarding treatment efficacy versus quality of life; these included: 1) situations in which they might consider stopping a treatment recommended by their clinician, and 2) whether they would give up an effective treatment with many side effects in favor of a less effective treatment with fewer side effects. We also asked participants to describe their experiences, if any, with treatment changes. All interviews were audio-recorded with participants’ permission and lasted approximately an hour.

### Data analysis

Descriptive statistics were used to summarize participants’ demographic characteristics. Participants’ narratives were analyzed using applied thematic analysis [23]. Interviews were transcribed verbatim following a transcription protocol [24], and NVivo version 12 [25](QSR International) qualitative data analysis software was used to organize the data and apply codes [26] to the transcripts. Trained qualitative analysts, who were also the interviewers, first independently applied structural codes based on the interview guide, segmenting participants’ interview narratives into broad categories related to the overall objectives (e.g., most important considerations for treatment). Inter-coder reliability (ICR) was assessed on 20% [27] of transcripts during the structural coding phase, discrepancies in code application were resolved through discussion, and any agreed-upon revisions to the structural coding were made. Next, the data were further subdivided into content codes, as the analysts identified themes within each structural code that reflected specific patient experiences and applied these to the transcripts. ICR assessments were conducted on 20% of transcripts during this stage of analysis as well, following the same procedure as before. After coding was completed, the analysts placed content codes into emergent thematic groups to identify common experiences among participant narratives and wrote analytical summaries of salient themes, including illustrative quotes. As no descriptive differences between Black and White participants were observed during analysis, all results thus reflect the interview sample as a whole. The COREQ checklist [28] informed the development of this manuscript.

### Ethics

The Duke University Health System Institutional Review Board (IRB) reviewed and approved the protocol (Pro00094133). All participants provided verbal informed consent.

## Results

### Study participants

We interviewed 21 people with multiple myeloma. Participants’ ages ranged from 36 to 78 years, 52% (*n* = 11) were female, and 38% (*n* = 8) were Black. All participants had graduated from high school, and 67% (*n* = 14) had a bachelor’s degree or higher. Most participants (*n* = 15; 71%) were retired or not currently working (Table 1). Fifteen participants (those recruited from CSC) self-reported having a physician diagnosis of multiple myeloma, while the six participants recruited from the transplant clinic had a confirmed diagnosis. Self-reported dates of diagnosis ranged from 21 years to 1 year prior to the interview; the majority (*n* = 14; 67%) were diagnosed within the previous 6 years. Slightly more than half (*n* = 11; 52%) of participants reported that they had experienced one or more relapses of multiple myeloma since diagnosis and treatment initiation, and 33% (*n* = 7) indicated that they had changed treatments.

**Table 1:** Participant demographics

Characteristic^a^	*N* (%) *n* = 21
**Age**	
35–44	1 (4.8)
45–54	3 (14.3)
55–64	10 (47.6)
65–74	6 (28.6)
75 +	1 (4.8)
**Sex**	
Female	11 (52.4)
Male	10 (47.6)
**Race**	
Black	8 (38.1)
White	12 (57.1)
Unknown	1 (4.8)
**Hispanic/Latino ethnicity**	
Not Hispanic or Latino	21 (100)
**Education**	
High school graduate	3 (14.3)
Some college or associate degree	4 (19.0)
Bachelor’s degree	5 (23.8)
Master’s or higher professional degree	9 (42.9)
**Employment status**	
Working part time	3 (14.3)
Working full time	3 (14.3)
Retired	11 (52.4)
Stay at home full-time	1 (4.8)
Disabled	3 (14.3)
**Marital status**	
Single	2 (9.5)
Married or domestic partnership	17 (81.0)
Divorced or separated	2 (9.5)
**Diagnosis**	
Self-report	15 (71.4)
Physician-confirmed	6 (28.6)
**Year of diagnosis**	
1999–2000	1 (4.8)
2001–2005	2 (9.5)
2006–2010	2 (9.5)
2011–2015	9 (42.9)
2016–2020	7 (33.3)

^a^Self-reported via online questionnaire or during screening

### Most important treatment considerations

Almost two-thirds of participants stated that their most important factor when considering treatment options was treatment efficacy. Participants explained that while it was important to them to balance quality of life impacts and other medical concerns to the greatest extent possible, they ultimately wanted a treatment that would be the best option to treat their cancer. A few participants specifically elaborated that their goal was to stay alive and expressed that they would be willing to tolerate adverse treatment effects if the treatment would allow them to live longer. See Table 2 for participant quotes.

**Table 2:** In-depth interview participant quotes on most important considerations for multiple myeloma treatment

**Topic**	**Participant quote**
Treatment efficacy	*Probably what was going to be the most efficacious. What was really going to work the best. …I mean, I wanted a medication that was going to work because my blood levels were very high and during that, if they didn’t go down quickly, I might have to go on kidney dialysis.…Basically, my concerns were getting what was going to work the quickest.* —White male, age 65, diagnosed in 2015 *The most important thing to me was living. …if I have to do it to live, I will do it. You know? If I have to do it, I will. I mean, like people say – I’ve heard people say they would never have another stem cell transplant. If it’s gonna prolong my life, yes, I will have another one, as horrible as it was. Yes.* —Black female, age 55, diagnosed in 2015
Quality of life	*And to me, the most important thing is to be able to function as normal as possible without any complications. I am still young enough that I have to work. I can’t do my job that I had. However, I can still function and work. …I don’t want to be classified as disabled or anything like that because then it cuts back all of your earning potentials and everything like that. So, everything is gridlocked. As long as the side effects and stuff don’t inhibit me being able to do my daily activities, I’m a happy camper.* —White male, age 60, diagnosed in 2016 *Quality of life is most important. As long as I’m still alive, I want my life to have quality and not be suffering the whole time.* —White male, age 69, diagnosed in 2012 *I was more – let’s get started and get to the part where I can be socially around folks again. …One of the qualities of life was being able to get around and visit people and go places and attend church and go to the shows and all the normal stuff that people do. …going on vacation and you know.* —Black male, age 60, diagnosed in 1999
Side effects of treatment	*Well, the additional consideration is myeloma screwed up my kidneys. So, when I choose a treatment, I have to make sure it doesn’t further injure my kidneys. That’s one consideration. …and to not increase the numbness in my feet.* —Female, no race specified, age 74, diagnosed in 2003
Trust in medical provider	*When my oncologist, hematologist, suggested a certain course of treatment, I just kind of went along with it. I asked other people, especially people in the myeloma support group. They concurred that it worked for this. And they really didn’t have a lot of side effects so basically I had to trust my doctor. That was the main thing. And since I’m not the expert, he is, and everything he suggested had worked, I felt like it was okay to follow his lead.* —Black female, age 55, diagnosed in 2015 *She's given me options, definitely. My doctor is great. She'll give me the side effects, and we kind of the least – trying to make it, the quality of life still good, but managing the disease.* —White female, age 52, diagnosed in 2005
Convenience	*I wanted a treatment that was not going to upset my daily life as much. A pill was pretty easy, but some of the treatments, you have to go into the [clinic] and get an infusion, and that would upset my schedule a little bit more, and seems more invasive. …So yeah, taking a pill, just to me, is the easiest thing, and that's what I wanted.* —White female, age 52, diagnosed in 2005
Multiple considerations are important	*I’m on the end of the scale that wanted what I thought and what the doctor thought was the best option to stop the bone lesions. So, the infusion drug that he is giving me has a side effect profile which is not particularly bad so I didn’t have a lot of hesitation in agreeing to go with that particular option. It seemed to me, based on the reading and my knowledge of the drugs that are currently available, his advice as the leading expert in the field, it seemed the best option for me so I went for it.* —White male, age 62, diagnosed in 2014
Changes in treatment considerations over time	*I think the only thing that’s different is I – the more drugs that are out there, the more I have learned, I would probably be willing to be more aggressive to maybe knock it into a deeper remission, you know, than I was six years ago. … I think it’s been a learning process for me of I used to think everything would be sequential as far as treatment. You’d go with whatever was the oldest in the pipeline, you’d go there, and then you’d take the next drug that came out, and the next, and kind of line them all up and go in that order. And you’d save the heavy hitters for maybe the very end. And now I’ve learned that we don’t need to save the heavy hitters for later. You know, you should use them now. So it’s not salvage therapy, it’s not desperation therapy, it’s use it now and get the best benefit. …and they’re less tolerable the worse shape you’re in, and the older you are, so use them now while you can tolerate them.* —White female, age 57, diagnosed in 2011

However, HRQoL remained an important consideration for participants. Just over half of participants stated that they also considered quality of life aspects such as the ability to function (described as being able to continue working and engaging in usual daily activities), the ability to enjoy life (including engaging in social and leisure activities, spending time with others, and being present for important life milestones), and not feeling sick (including both minimization of treatment-related side effects and alleviation of multiple myeloma-related symptoms), when considering potential multiple myeloma treatments. In particular, participants raised concerns about the possibility of experiencing treatment-related side effects, with nearly a third describing this as an important factor in their thinking. For example, participant worries included the potential for fatigue and mental fog that might make it difficult for them to function, or the possibility that treatment might damage other organ systems such as kidneys that had already been compromised by the multiple myeloma.

Some participants described that while they considered a variety of factors when selecting a cancer treatment, the most convincing argument in favor of a particular treatment was ultimately their hematologist/oncologist’s recommendation. These participants elaborated that they trusted their clinician to understand what was important to them and help them strike the right balance between side effects and efficacy. They explained that the clinician was the expert, they perceived the clinician as having their best interests at heart, and that treatments previously recommended by the clinician had proven effective. Convenience and ease of treatment administration also factored into a few participants’ decision-making, as they described that making frequent return visits to the clinic was disruptive to their schedule, particularly for those who were trying to return to work. Thus, these participants preferred less-frequent treatment regimens or would prefer to receive treatment in a form (e.g., orally) that could be administered at home without the presence of medical personnel.

While some participants described a single primary consideration that influenced their treatment decision-making, most explained that they considered and weighed a variety of personally important factors when choosing a multiple myeloma treatment. This pattern continued when participants were asked how their treatment considerations had changed over time, with most describing that they continued to consider multiple factors. However, while a few noted no change to their treatment considerations, most described changes in the relative importance of different factors. For example, a participant expressed that over time, quality of life considerations and convenience of treatment had taken on greater significance for them, while another noted that as time passed, they had become willing to be more aggressive with treatment in order to have a better chance of eradicating the cancer.

### Tradeoffs

More than half of participants indicated that they would stop a clinician-recommended multiple myeloma treatment if it would negatively affect their quality of life. “Concerns” or “reasons to stop” included intolerable side effects such as feeling excessively ill, adverse treatment-related impacts on participants’ ability to function, and the potential for the treatment to cause future harm, such as development of other cancers, or damage to organ systems. A few participants also discussed that they would consider treatment efficacy in this scenario, noting both positive and negative considerations that might lead them to stop treatment, including if a better treatment became available or conversely, if prognosis was poor despite the treatment. See Table 3 for participant quotes.

**Table 3:** In-depth interview participant quotes on tradeoff considerations between efficacy and side effects

**Topic**	**Participant quote**	
Situations in which participants would stop a recommended treatment	Intolerable side effects	*Ooh. That’s a tough question. I don’t know. I really have faith in what he recommends. Obviously, I’m not a doctor. So, if he thinks that this is going to be the best thing to get my numbers into a good place, then I want to follow what he’s going to say. But obviously, if I was deathly ill because of the medication or completely nauseated or something all the time, I’m sure I would want to put a halt to that and try to find – because with multiple myeloma, there’s – if A doesn’t work, we try B. If B doesn’t work, we try C. And if I’m uncomfortable with C, let’s move on to D.* —White male, age 60, diagnosed in 2016
	Adverse impact on functioning	*Probably only if it would really make things non-functional. …If I didn’t get up. If I couldn’t be about my daily life. …I mean, now sometimes I’ll still have to make adjustments, but I’m still moving forward and going on with it. Sometimes I have to sit down more and that type of thing. But I’m still going.* —White female, age 50, diagnosed in 2016
	Treatment causes harm	*Or if I found that the risk of the side effects was greater than the benefit in terms of my heart or lungs, or something like that.* —White female, age 64, diagnosed in 2014
	Treatment efficacy	*That’s a tough one. Because I don’t want to stop any treatment unless there’s a better treatment. Yeah. I can’t honestly answer that one. Unless there’s a better treatment, I’m not gonna stop it and do nothing.* —Black female, age 55, diagnosed in 2015
Willingness to trade an effective treatment for a treatment with fewer side effects	Preference for most efficacious treatment	*No. I want the best treatment that’s available because I wouldn’t want to take the risk. It feels like to me that I don't know enough medically, but I wouldn’t want to take the risk of the cancer surfacing for an easier treatment because that may be fatal. And unless somebody could assure me that it’s not fatal, but why would I want the discomfort, right? So, I just don’t know why I would do that. …It’s like okay, I’ll take short-term over long-term. Yeah, I don't want to do that.* —Black male, age 67, diagnosed in 2014
	Treatment efficacy	*I guess just you don't want the disease to be progressing, because it can cause so many more problems, so you have to balance that. If it was working less, but still slow, if it was still kind of keeping me at bay, then I would definitely consider it, but if my PET scans were getting worse, then I would probably just go back and deal with the side effects, because like I said, once you have this stuff happen with your bones and your organs, it’s hard to go backwards. You don't want to have a fracture or have kidney problems and go on dialysis. You don't want the future problems, and that could happen if the medicine wasn’t working completely. So I guess it depends on the medicine; how much is it working? Is it working a little bit, or is it working pretty good?* —White female, age 52, diagnosed in 2005
	Severity of side effects	*I think for me, it would depend on how bad are the side effects with the treatment that’s working. Are they so bad that I just can’t tolerate it anymore, in which case I’d surely guess I would stop. If they were bad but I was still getting decent efficacy out of the drug, I think I would hang on.* —White male, age 62, diagnosed in 2014
	Duration of side effects	*It depends on the duration of the treatment. …So, if I was going to be on a treatment that for three months, I would be miserable, but it was supposed to be more effective than possibly another med, I could live three months miserable, if it was going to be effective vs. this is how it’s going to be for the next year. I would have to evaluate the timeline of what it was going to be like. …Yeah, three to four months, probably. I could be – and then, I would have to reevaluate it. Can I do this another three months? …So, I think four months is kind of my – three to four months is kind of my okay, let’s see where we are and regroup.* —White female, age 62, diagnosed in 2016

Most participants expressed that they would not trade a more efficacious treatment for one that had fewer side effects but was less effective, echoing participants’ earlier statements about prioritizing treatment efficacy. Participants described wanting the best option available to treat the cancer, not wanting to interfere with a treatment that was working for fear the change might cause the multiple myeloma to come back, and not wanting to experiment if they were able to effectively manage side effects. Some participants also discussed wanting to avoid long-term myeloma-related damage that might occur with a less effective treatment, noting that if a treatment were not strong enough to keep all of the adverse effects of multiple myeloma at bay, they might sustain lifelong damage to bones or kidneys as a result of choosing a treatment with fewer side effects. A few participants stated definitively that they would not be willing to change from a treatment that was working or that they might even be willing to accept a treatment with a worse side effect profile if that treatment were also more effective or aggressive.

However, some participants tempered their comments by stating that their willingness to stay on the most effective treatment would depend on the severity of side effects experienced. These participants would be more willing to change to a less effective treatment if they were experiencing intolerable side effects or if the more effective treatment had a high risk of harmful side effects. The duration of treatment also factored into some participants’ thinking. Several described that they would be willing to tolerate aversive treatment effects for a period of up to several months, but none volunteered that they would be willing to experience these effects for as long as a year. Participants discussed that they would engage in a continual process of re-evaluation after a certain time point, to determine whether they felt able to continue with the treatment.

### Experience with treatment changes

More than half of participants stated that they had not asked or thought about asking their hematologist/oncologist to change their myeloma treatment, largely because their current treatment was working or they trusted their clinician’s recommendation about the most appropriate therapy. Some also described that they had not made a treatment change out of worry that changing treatments would cause progression or relapse of their disease. However, around a third of participants indicated that they had experienced a treatment change. Further, a few noted that although they had not yet changed treatments, the impending need to do so was an ongoing topic of conversation with their clinician. See Table 4 for participant quotes.

**Table 4:** In-depth interview participant quotes on experience with treatment changes

**Topic**	**Participant quote**	
Has not made a treatment change	*I’ve been to blood cancer conferences and people talk about taking a medication holiday, where you’re coming off the medicine for a period of time, and the doctors say, yeah, you can take a holiday from the medicine as long as your numbers are good and everything. And I’m like, you know what, I’m not touching it. I’m gonna take the maintenance and just keep taking it until – I’m afraid to even take that medication holiday because I’m just like, maybe if I stop taking it for a while, then the multiple myeloma is just gonna come back. It’s just gonna rear its ugly head so I’d rather – to take that pill, it really doesn’t affect me that much to take a pill. I don’t have a problem swallowing pills. So, as long as the doctor told me to do it, I trust him. I’ve seen good results. I’m still here. So, I’m not gonna mess with anything.* —Black female, age 55, diagnosed in 2015	
Actively discussing prospective treatment change	*Well, we are discussing it now because my numbers are starting to move upward. So we’ve been discussing when, you know, at what level we would wait to change and then what we would change to. So we have been discussing that, but because it’s so well tolerated up until now and working, we haven’t had any reason – to me, I felt no reason in discussing changing treatment. But now we are because we know my numbers are increasing, and eventually I’m going to have to. So, we like to look down the road.* —White female, age 57, diagnosed in 2011	
Has made a treatment change	Treatment stopped working	*When the treatment doesn’t work is when I have to – they change it, but the oncologist knows damn well it’s not working, so they’d better change to try something else. There’s no cure for this damn disease, but there are treatments, and there’s new treatments coming up all the time, so, thank God I can move to a different treatment when the current one stops working. …So, the current treatment, where we’ve reduced the dosage and reduced the frequency from weekly to biweekly – so far, knock wood, that has been working, and I’m hoping it’s gonna continue working. …You keep on moving to other treatments until you run out of options, and then, thank God the FDA’s approving drugs in the meantime to give a go.* —White male, age 69, diagnosed in 2012
	Treatment change due to side effects	*If [side effects are] too dominating in my life, I just stop the meds for a while and I tell my doctor. …Once, I asked to stop for a year and they said no. …That was rather dramatic. They said six months, and we proceeded to do that, and after three months, my marker doubled, and I went back on the meds. After three months of being off them, yeah. I don’t want to be stupid about this. So I went right back on. …I usually just ask to stop. I’m not a fan of switching meds when the ones that I’m taking are doing what I want, but I’m a fan of stopping them to let my body recover and then keep going with the same ones. It’s kind of a practical choice rather than starting something new.* —Female, no race specified, age 74, diagnosed in 2003 *I think the protocol when she first started me on it is just 25 mg, so that’s what we went on, and it was kind of a newer medicine, so we just went with 25. And then after some clean PET scans over a couple of years we reduced it to 15, and it was causing some stomach problems, and then at 15, it was fine. And then after probably two more PET scans that were good, she's like, let's try 10. But then we upped it back to 15 last year, just because there’s a couple of things from the PET scan that are very minor, but she just wants to make sure. So we're back at 15 now. …Actually, I think it was my suggestion. Only initially, from 25 to 15, was because I was having stomach problems, and she was like, okay, well, here’s what we can try. So it was actually me initiating it, because of the side effects. And then when we went to 10, I was kind of initiating it, too, and she agreed. And then going back to 15 was kind of her suggestion last year.* —White female, age 52, diagnosed in 2005
	Change in mode of administration	*I mentioned initially, my first treatment was two oral drugs and a subcutaneous injection. The subcutaneous injection, I used to get rashes at the site of the injection and that subcutaneous was twice a week. It was not particularly painful but it actually became difficult for them to find sites to inject with my going back in there so frequently. So, there was an oral version of this drug that works the same way as the subcutaneous one, and so I asked him can we switch to this one. If it’s oral, I’m not going to get an injection site reaction, it’s more convenient for me because I’m not going to the clinic for a sub-cu shot, and so we did. We actually switched from the subcutaneous to the oral drug that works by the same mechanism.* —White male, age 62, diagnosed in 2014

Treatment changes were often necessitated by signs that a medication had stopped working or was becoming less effective at suppressing the myeloma. Participants also discussed making treatment changes in response to side effects. The most commonly described changes were dosage adjustments or switching to a different myeloma treatment; a few participants noted that they had experienced both types of changes. Side effects also led to temporary or permanent treatment stoppage for a few participants, who discussed that they had taken a break or stopped treatment because of adverse reactions. A few participants additionally described that their preferences for oral routes of administration or decreased administration frequency led to a treatment change.

## Discussion

Our findings reflect factors and considerations that are important to patients undergoing multiple myeloma treatment in the US. The study sample included racially diverse participants, as well as those representing different stages of the disease trajectory. Participants emphasized the primary importance of treatment efficacy and their desire for the best option to treat the cancer, yet their narratives also highlighted concern that effective treatment might entail sacrificing aspects of HRQoL. The need to balance efficacy with quality of life, finding a treatment that allows for good myeloma control while also enabling as much normal functioning as possible, was commonly expressed. Participants shared that they wished to avoid future harm from myeloma and its treatments, did not want to feel sick or experience other aversive side effects, and did not want to be unduly burdened by the mode or frequency of treatment administration. Participants also expressed that their hematologist/oncologist’s treatment recommendation served as an important consideration and that they trusted their clinician to suggest treatments that reflected their values.

Similar findings to ours have been obtained in European and Canadian patient samples, in studies using a variety of methods to elicit treatment preferences from myeloma patients. Several studies have highlighted the importance to patients of progression-free survival and increased life expectancy [15, 17, 29, 30]. For example, Mühlbacher and colleagues conducted focus groups for concept elicitation followed by a survey of 282 patients in which the relative importance of various multiple myeloma therapy characteristics was assessed via both Likert scale and DCE; on both measurements, patients ranked elements related to treatment efficacy and prolonged life expectancy as most important [29]. In another study that used an online survey of 560 patients to examine preferences for multiple myeloma treatment attributes, Postmus and colleagues found that increasing the probability of surviving progression-free for a year or longer was weighted more strongly by patients than was the probability of decreasing either mild/moderate chronic toxicity or severe/life-threatening toxicity associated with treatment [30].

Other qualitative interview studies with myeloma patients have also reported on the tension many patients experience between treatments that prolong life but produce disruptive side effects [15, 17, 18]. A group of Canadian patients who were interviewed about their experiences living with relapsed or refractory multiple myeloma expressed that while life expectancy was one of their highest treatment priorities, physical and cognitive side effects related to treatment were also significant for their potential impact on functioning and quality of life, and as with our sample, some patients in this study described that their treatment priorities changed over time [17]. A recent focus group study of patients in four countries [18], seeking to identify key attributes impacting myeloma patients’ treatment choices, also described participants’ perspectives on the importance of balancing life expectancy with the experience of side effects that could negatively impact quality of life. Patients in this study echoed concerns voiced by our sample about trading short-term benefits for long-term negative effects and expressed that duration and severity of side effects might induce them to consider a treatment change.

Our findings also support data from other studies [15, 31] showing that frequency and mode of administration are important considerations for myeloma patients. A DCE study of relapsed refractory myeloma patients in Germany found that mode of administration was considered an important aspect of patients’ quality of life; specifically, these patients preferred oral treatment that avoided the need for them to undergo lengthy in-clinic infusions [31]. Patients in another recent multi-country interview study also described burden due to duration of infusions and travel time to the clinic; these patients expressed a preference for in-home treatments, where available [15].

In our sample, a few participants described engaging in ongoing dialogue with their hematologist/oncologist regarding future treatment changes, illustrating the shared decision-making (SDM) approach to treatment choice; participants also expressed trust that their clinician’s judgment about the most appropriate treatment would reflect their values. Unfortunately, studies have shown that many clinicians may not follow an SDM framework in clinical practice, instead falling back on one-sided informational presentations [32]. Our findings highlight the importance of operationalizing and following an SDM process with multiple myeloma patients, thus ensuring that patients’ goals and preferences are utilized to inform clinicians’ treatment recommendations.

Our study links to many others in the literature that have reported on the treatment-related benefits, quality of life impacts, and burdens that matter most to multiple myeloma patients, confirming and extending these findings to a US-based patient population that intentionally included non-White participants. The racial diversity of our sample, as well as its heterogeneity with respect to disease stage and treatment experience, represent strengths of our study. However, the limitations of our research must also be considered. As a qualitative study with 21 participants that were purposefully sampled, a different group of participants may have shared different experiences. Further, the self-selection of much of our sample from an online panel of cancer patients may have resulted in a sample that was more engaged and knowledgeable about treatment options than myeloma patients at large. Finally, participants self-reported their disease history, and some were not able to describe the specific details about their cancer course and multiple treatments. This is mitigated, however, by the fact that our focus was on treatment considerations and experiences that apply to myeloma patients generally, without reference to specific disease stage or relapse history.

## Conclusions

In conclusion, our qualitative interviews on participants’ experiences with multiple myeloma treatment and the considerations that are most important to them revealed that while participants placed high value on treatment efficacy, maintaining quality of life was of almost equal importance. Participants’ tradeoff considerations were driven by the desire to maintain health and functioning, avoid future harm, minimize adverse treatment effects, and maximize convenience. Participants also expressed trust in their hematologist/oncologist’s recommendations and viewed their clinicians as well positioned to understand and reflect their values when suggesting treatments for multiple myeloma. Operationalizing patients’ treatment preferences via a shared decision-making framework is recommended.

## Data Availability

The datasets used and/or analysed during the current study are available from the corresponding author on reasonable request.

## Appendix

Selected questions from the in-depth interview guide with multiple myeloma patients that informed this analysis and manuscript

**Table:**

**Section ****1: Treatment(s) and treatment goals**
*Objective: This section allows us to characterize patients’ treatment trajectory since diagnosis and determine key factors that influenced treatment decision making over time.*

When thinking about your treatment(s) [*if relevant:* after your multiple myeloma came back], what factors or considerations were most important to you? For example, some people might want a treatment that has fewer side effects or symptoms, even if it’s not as effective as other treatments. Whereas others might want the most effective cancer treatment, even if it typically has a lot of side effects or symptoms. *Probe until all factors and considerations are mentioned and probe about reasons why those factors were important – e.g., *“*Starting with your first round after your cancer came back… What about your current round of treatment?”*

You mentioned [repeat factors/considerations] as influences on your thinking about the type of treatment that is best for you. How have these factors or considerations changed over time?

In what situations would you possibly consider stopping a treatment that your physician recommended? *Probe about if the symptoms got worse*

If your symptoms get worse, would you be willing to give up a treatment that is working, in favor of a treatment with fewer symptoms but that may not work as well?

Have you ever asked—or thought about asking—your medical provider to change treatments? If so, please describe. *Probe about whether actually asked or just thought about asking, reasons asked for a change, provider’s response, and satisfaction with change*
